# Assessment of the Acceptability and Feasibility of Using Mobile Robotic Systems for Patient Evaluation

**DOI:** 10.1001/jamanetworkopen.2021.0667

**Published:** 2021-03-04

**Authors:** Peter R. Chai, Farah Z. Dadabhoy, Hen-Wei Huang, Jacqueline N. Chu, Annie Feng, Hien M. Le, Joy Collins, Marco da Silva, Marc Raibert, Chin Hur, Edward W. Boyer, Giovanni Traverso

**Affiliations:** 1 Department of Emergency Medicine, Brigham and Women’s Hospital, Boston, Massachusetts; 2 Department of Psychosocial Oncology and Palliative Care, Dana Farber Cancer Institute, Boston, Massachusetts; 3 The Fenway Institute, Boston, Massachusetts; 4 The Koch Institute for Integrated Cancer Research, Massachusetts Institute of Technology, Cambridge; 5 Division of Gastroenterology, Department of Medicine, Brigham and Women’s Hospital, Boston, Massachusetts; 6 Division of Gastroenterology, Department of Medicine, Massachusetts General Hospital, Boston; 7 Department of Electrical Engineering and Computer Science, Massachusetts Institute of Technology, Cambridge; 8 Boston Dynamics, Waltham, Massachusetts; 9 Division of Gastroenterology, Department of Medicine, Columbia University, New York, New York; 10 Department of Mechanical Engineering, Massachusetts Institute of Technology, Cambridge

## Abstract

**Importance:**

Before the widespread implementation of robotic systems to provide patient care during the COVID-19 pandemic occurs, it is important to understand the acceptability of these systems among patients and the economic consequences associated with the adoption of robotics in health care settings.

**Objective:**

To assess the acceptability and feasibility of using a mobile robotic system to facilitate health care tasks.

**Design, Setting, and Participants:**

This study included 2 components: a national survey to examine the acceptability of using robotic systems to perform health care tasks in a hospital setting and a single-site cohort study of patient experiences and satisfaction with the use of a mobile robotic system to facilitate triage and telehealth tasks in the emergency department (ED). The national survey comprised individuals living in the US who participated in a sampling-based survey via an online analytic platform. Participants completed the national survey between August 18 and August 21, 2020. The single-site cohort study included patients living in the US who presented to the ED of a large urban academic hospital providing quaternary care in Boston, Massachusetts between April and August 2020. All data were analyzed from August to October 2020.

**Exposures:**

Participants in the national survey completed an online survey to measure the acceptability of using a mobile robotic system to perform health care tasks (facilitating telehealth interviews, acquiring vital signs, obtaining nasal or oral swabs, placing an intravenous catheter, performing phlebotomy, and turning a patient in bed) in a hospital setting in the contexts of general interaction and interaction during the COVID-19 pandemic. Patients in the cohort study were exposed to a mobile robotic system, which was controlled by an ED clinician and used to facilitate a triage interview. After exposure, patients completed an assessment to measure their satisfaction with the robotic system.

**Main Outcomes and Measures:**

Acceptability of the use of a mobile robotic system to facilitate health care tasks in a hospital setting (national survey) and feasibility and patient satisfaction regarding the use of a mobile robotic system in the ED (cohort study).

**Results:**

For the national survey, 1154 participants completed all acceptability questions, representing a participation rate of 35%. After sample matching, a nationally representative sample of 1000 participants (mean [SD] age, 48.7 [17.0] years; 535 women [53.5%]) was included in the analysis. With regard to the usefulness of a robotic system to perform specific health care tasks, the response of “somewhat useful” was selected by 373 participants (37.3%) for facilitating telehealth interviews, 350 participants (35.0%) for acquiring vital signs, 307 participants (30.7%) for obtaining nasal or oral swabs, 228 participants (22.8%) for placing an intravenous catheter, 249 participants (24.9%) for performing phlebotomy, and 371 participants (37.1%) for turning a patient in bed. The response of “extremely useful” was selected by 287 participants (28.7%) for facilitating telehealth interviews, 413 participants (41.3%) for acquiring vital signs, 192 participants (19.2%) for obtaining nasal or oral swabs, 159 participants (15.9%) for placing an intravenous catheter, 167 participants (16.7%) for performing phlebotomy, and 371 participants (37.1%) for turning a patient in bed. In the context of the COVID-19 pandemic, the median number of individuals who perceived the application of robotic systems to be acceptable for completing telehealth interviews, obtaining nasal and oral swabs, placing an intravenous catheter, and performing phlebotomy increased. For the ED cohort study, 51 individuals were invited to participate, and 41 participants (80.4%) enrolled. One participant was unable to complete the study procedures because of a signaling malfunction in the robotic system. Forty patients (mean [SD] age, 45.8 [2.7] years; 29 women [72.5%]) completed the mobile robotic system–facilitated triage interview, and 37 patients (92.5%) reported that the interaction was satisfactory. A total of 33 participants (82.5%) reported that their experience of receiving an interview facilitated by a mobile robotic system was as satisfactory as receiving an in-person interview from a clinician.

**Conclusions and Relevance:**

In this study, a mobile robotic system was perceived to be acceptable for use in a broad set of health care tasks among survey respondents across the US. The use of a mobile robotic system enabled the facilitation of contactless triage interviews of patients in the ED and was considered acceptable among participants. Most patients in the ED rated the quality of mobile robotic system–facilitated interaction to be equivalent to in-person interaction with a clinician.

## Key Points

**Question:**

Is the use of a mobile robotic system to evaluate patients in the emergency department acceptable and feasible?

**Findings:**

In this survey and cohort study comprising a national survey of 1000 participants across the US and a single-site cohort of 40 patients presenting to the emergency department, 93% of participants reported that their experience of interacting with a mobile robotic system was satisfactory, and most participants believed that using a robotic system for facilitating health care tasks was acceptable. A total of 83% of participants reported that their experience with a robotic system–facilitated triage interview in the emergency department was equivalent in quality to an in-person interview conducted by a clinician.

**Meaning:**

In this study, the use of a mobile robotic system was perceived as satisfactory and acceptable for the facilitation of health care tasks in a hospital setting.

## Teaser

This descriptive survey and cohort study investigates the acceptability and feasibility of using a mobile robotic system to facilitate health care tasks in a hospital setting.

## Introduction

The coronavirus disease 2019 (COVID-19) pandemic has changed the manner in which clinicians interact with patients. Personal protective equipment, social distancing, and triage facilities to screen symptomatic individuals have been implemented to protect health care professionals and prevent transmission of severe acute respiratory syndrome coronavirus 2 (SARS-CoV-2).^1,2,3,4,5,6^ Despite these measures, health care professionals continue to be at high risk for COVID-19; one study reported that up to 20% of infections in Italy were among health care professionals.^7^ Clinicians who acquire COVID-19 are unable to provide direct patient care, thereby decreasing the availability of an essential workforce during the pandemic.^8,9^

While the development of pharmacotherapies and vaccines to address COVID-19 continues to advance, many health care systems have expanded their telehealth capabilities with the aim of limiting human contact while permitting triage of patients who may have COVID-19 .^3,10^ These solutions enable clinicians to deliver care virtually, determine the need for additional testing, and conduct follow-up visits in a contactless manner.^11,12^

Many existing telehealth platforms rely on static patient-controlled tablet computers or smartphones. The use of mobile robotic telehealth systems controlled by clinicians can facilitate a dynamic evaluation process that can be used in the hospital setting.^13^ Placed on a robotic chassis, these telehealth systems can facilitate evaluation of patients in various settings.^14^ Robotic systems represent a mobile telepresence that can move between patients, rooms, or wards within a hospital setting.^15^ In field hospitals erected to manage the influx of patients with COVID-19, the use of an agile robotic system may obviate the need to install temporary static infrastructure to support traditional telehealth systems.^16^ Before the widespread implementation of robotic systems to provide patient care during the COVID-19 pandemic occurs, it is important to understand the acceptability of these systems among patients and the economic consequences associated with the adoption of robotics in health care settings.^17^ In this investigation, we sought to understand attitudes toward robotic system–facilitated health care tasks, such as the facilitation of telehealth interviews and the acquisition of contactless vital signs and nasal and oral swabs, among a national sample of individuals in the US. In addition, we used a mobile robotic system to facilitate contactless triage interviews of patients with potential COVID-19 in the emergency department (ED).

## Methods

This study consisted of 2 components: (1) a national sampling-based survey of individuals across the US to examine the acceptability of using robotic systems to facilitate health care tasks in a hospital setting and (2) a single-site cohort study of patient experiences and satisfaction with the use of a mobile robotic telehealth system to facilitate triage and telehealth tasks in the ED of a large urban academic hospital providing quaternary care in Boston, Massachusetts during the COVID-19 pandemic. The study was approved by the institutional review board of Mass General Brigham. All patients in the ED cohort study provided verbal informed consent, and all participants in the national survey provided digital informed consent. This study followed the Strengthening the Reporting of Observational Studies in Epidemiology (STROBE) reporting guideline for cohort studies.

### National Survey

We partnered with a global market research and data analytics service (YouGov) to conduct a national survey on attitudes about the acceptability of using robotic systems in hospital settings among US residents. We developed a survey questionnaire (eMethods in the Supplement) that was based on the Negative Attitudes Toward Robots Scale (NARS), a quantitative measure that evaluates attitudes toward robotic systems.^18,19^ Survey responses were measured using a 5-point Likert scale (with 1 indicating strongly disagree, 2 indicating disagree, 3 indicating neither agree nor disagree, 4 indicating agree, and 5 indicating strongly agree). We also developed questions regarding respondents’ perceptions of the usefulness of robotic systems to facilitate specific health care tasks, such as facilitating a telehealth interview, acquiring contactless vital signs, obtaining a nasal or oral swab, placing an intravenous catheter, performing phlebotomy, and turning a patient in bed. Question responses were based on a 5-point scale (with 1 indicating extremely useless, 2 indicating somewhat useless, 3 indicating neither useful nor useless [neutral], 4 indicating somewhat useful, and 5 indicating extremely useful). We specifically did not provide images or descriptions of robotic systems because we wanted respondents to consider their general perceptions of the use of robots in a health care setting. We first asked these questions in the context of general interaction with robotic systems in the hospital. Next, we asked participants to consider the usefulness of robotic systems in the context of the COVID-19 pandemic, with an emphasis on using robotic systems to limit direct human contact and conserve personal protective equipment.

Participants completed surveys from August 18 to August 21, 2020. We obtained informed consent using a fact sheet approved by the institutional review board, which was presented to all potential participants. Consenting participants acknowledged the fact sheet, provided verbal consent, and were presented with the survey on the analytics platform (YouGov). Because this platform conducts sampling using an opt-in panel of participants, the survey format was defined as a nonprobability internet panel following the American Association for Public Opinion Research (AAPOR) reporting guideline.^20^ The participation rate was calculated, raw results were tabulated, and weights were applied to ensure representation of a national sample. We measured a composite NARS score among study participants using the NARS S1 subscale, which assesses baseline negative attitudes toward robotic systems.

We calculated basic descriptive statistics (mean, SD, and minimum and maximum values) to characterize NARS scores among participants. For questions considering the usefulness of robotic systems to facilitate specific health care tasks, we calculated basic descriptive statistics (median and interquartile range [IQR]) to compare usefulness scores within the contexts of general interaction and interaction during the COVID-19 pandemic in a hospital setting. We used the Wilcoxon signed rank test to compare the responses between these 2 contexts and assess whether the differences were statistically significant.

### Cohort Study

**Figure 1.**
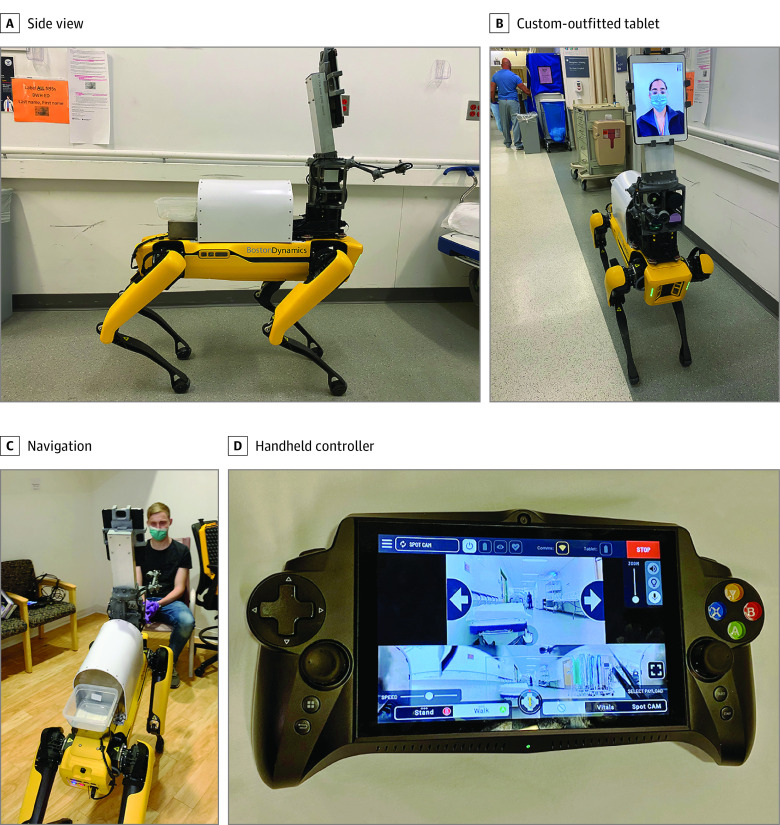
Quadruped Robotic System A, Side view of the quadruped robotic system (Dr Spot). B, Custom-outfitted tablet for mobile telehealth interviews of patients in the emergency department. C, Navigation. A trained operator navigates the robotic system to a patient to remotely conduct triage. D, Handheld controller for mobile robotic system.

We conducted a single-site cohort study to examine feasibility and acceptability of the use of a robotic system to facilitate telehealth triage within the ED setting during the COVID-19 pandemic. The study was conducted from April to August 2020 in the ED of Brigham and Women’s Hospital, which evaluates approximately 60 000 patients annually. We used an agile quadruped robotic system (Dr Spot; Boston Dynamics) to perform contactless triage interviews (Figure 1).^21^

We enrolled adult patients presenting to the ED who were triaged in the novel tent space or the standard ED waiting room or who directly received a room in the ED. All enrolled patients were medically stable and able to participate in an interview. Potential participants were approached by a member of the study team on a convenience basis. After the study procedures were described to potential participants, they were asked questions about those procedures to ensure their understanding. Individuals then provided verbal consent to participate. For those who did not speak English, a certified hospital interpreter explained the study procedures and obtained informed consent. Next, participants were exposed to the robotic teletriage system, which was controlled by a trained emergency medical professional. The clinician navigated the robot through the ED to the participant’s location and conducted a triage interview via the integrated video link on the tablet computer. At the conclusion of the encounter, participants completed a quantitative assessment based on the Telehealth Usability Questionnaire, in which they were asked to rate whether they were dissatisfied, neutral, or satisfied with their experience with the robotic system.^22^ After each patient encounter, the robotic system chassis was sterilized with ethanol wipes. Basic descriptive statistics were calculated to describe participant responses.

### Design of Mobile Robotic System

The mobile robotic system consisted of a 4-legged robot outfitted with a secure communication relay to a tablet controller, which allowed a single operator to navigate the robot Video). We initially used the robotic system as a WiFi access point, with the robot outfitted as a 2.4-GHz access point linking the robot to a handheld controller. This technique was successful in maintaining control of the robot; however, during preliminary testing in the ED, increased congestion of wireless radio bands from patients’ smartphones and other connected devices within the ED produced frequent signal loss if the operator did not have a clear line of sight to the robot. To allow the operator to remain at a static location in the ED, we switched to a mesh radio system, which consisted of an on-board radio payload (Rajant Corp) attached to the robot and a receiver attached to the operator. This radio system used 2.4-GHz and 5.8-GHz bands, thereby avoiding interference from conventional systems that used the Institute of Electrical and Electronics Engineers 802.11 WiFi standard. Both the robot as a WiFi access point and the mesh radio system had bandwidths of 0.5 to 2.5 megabits per second to carry command and control signals as well as video streams.

We also outfitted the robot with a tablet computer, which ran a real-time person-to-person video link that allowed us to conduct telehealth interviews in the ED. Video and data transmissions from the robot to the operator were encrypted at each end based on transport layer security standards. We conducted a standardized training program to instruct emergency medical professionals (physicians and physician assistants) in the operation of the robotic system and tablet computer. Emergency medical professionals were asked to perform an initial triage interview (ie, obtain a patient history) via the robotic system.

### Statistical Analysis

All data analysis was completed using Stata software, version 16.1 (StataCorp). Data were analyzed from August to October 2020.

## Results

### National Survey on Acceptability

A total of 3223 individuals were invited to participate in the national acceptability survey. Among those, 1339 distinct surveys were initiated, and 1154 surveys were completed, representing a participation rate of 35%. After data collection, sample matching was performed to generate a nationally representative group of 1000 respondents who were distributed across the US. The mean (SD) age of participants was 48.7 (17.0) years; 535 participants (53.5%) were female, and 465 participants (46.5%) were male (eTable 1 in the Supplement). A total of 719 participants (71.9%) were White, and 677 participants (67.7%) had attended college, received a 2-year or 4-year college degree, or attended graduate school. The mean (SD) NARS S1 score among participants was 16.3 (4.8) points,^19^ which was within the lower range of NARS S1 scores and indicated that the study population was relatively accepting of interactions with robotic systems.^23,24^

**Figure 2.**
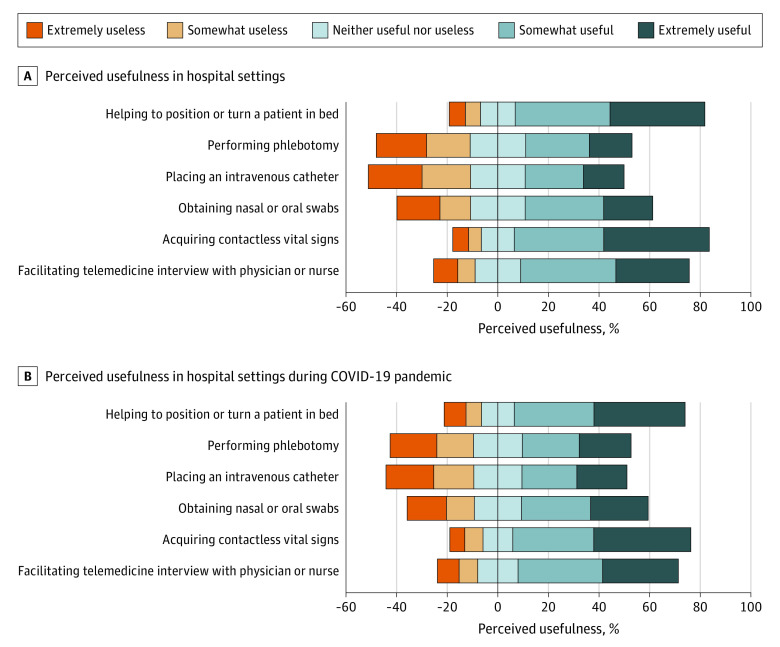
Participant Responses to National Survey Regarding Perceived Usefulness of Robotic Systems to Facilitate Common Health Care Tasks A, Perceived usefulness in hospital settings. B, Perceived usefulness in hospital settings during COVID-19 pandemic. COVID-19 indicates coronavirus disease 2019.

We selected 6 questions that reflected health care tasks with which robotic systems may assist during the COVID-19 pandemic: facilitating a telehealth interview, acquiring contactless vital signs, placing an intravenous catheter, performing phlebotomy, obtaining nasal and oral swabs, and turning a patient in bed from their back to their abdomen (ie, proning) (Figure 2). With regard to the usefulness of a robotic system to perform specific health care tasks, the response of “somewhat useful” was selected by 373 participants (37.3%) for facilitating telehealth interviews, 350 participants (35.0%) for acquiring vital signs, 307 participants (30.7%) for obtaining nasal or oral swabs, 228 participants (22.8%) for placing an intravenous catheter, 249 participants (24.9%) for performing phlebotomy, and 371 participants (37.1%) for turning a patient in bed. The response of “extremely useful” was selected by 287 participants (28.7%) for facilitating telehealth interviews, 413 participants (41.3%) for acquiring vital signs, 192 participants (19.2%) for obtaining nasal or oral swabs, 159 participants (15.9%) for placing an intravenous catheter, 167 participants (16.7%) for performing phlebotomy, and 371 participants (37.1%) for turning a patient in bed (eTable 2 in the Supplement).

Median scores for the usefulness of tasks performed in a hospital setting were neutral (ie, rated as neither useful nor useless) with regard to placing an intravenous catheter (3 points; IQR, 2-4 points), performing phlebotomy (3 points; IQR, 2-4 points), and obtaining nasal and oral swabs (3 points; IQR, 2-4 points). Median scores were higher (ie, rated as somewhat useful) with regard to facilitating telehealth interviews (4 points; IQR, 3-5 points), acquiring contactless vitals (4 points; IQR, 4-5 points), and assistance with turning a patient in bed (4 points; IQR, 3-5 points). When asked to consider the use of robotic systems to perform these same tasks in the context of the COVID-19 pandemic, the median score for obtaining nasal and oral swabs changed from neutral (3 points; IQR, 2-4 points) to somewhat useful (4 points; IQR, 2-4 points). Other median scores were unchanged (eTable 2 in the Supplement).

Although median usefulness scores did not change for most tasks, the Wilcoxon signed rank test indicated that a statistically significant number of individuals changed their usefulness ranking for robotic system–facilitated tasks in the context of the COVID-19 pandemic. For example, more respondents considered the robotic system to be extremely useful in the context of interaction during the pandemic vs general interaction in the hospital setting for the tasks of placing an intravenous catheter (208 participants [20.8%] vs 159 participants [15.9%], respectively; *P* < .001), performing phlebotomy (215 participants [21.5%] vs 167 participants [16.7%]; *P* < .001), obtaining a nasal or oral swab (239 participants [23.9%] vs 193 participants [19.3%]; *P* = .002), and turning a patient in bed (378 participants [37.8%] vs 371 participants [37.1%]; *P* = .04). No significant change was observed in the usefulness of robotic systems for facilitating telehealth interviews (eTable 2 in the Supplement).

### Cohort Study of Satisfaction 

A total of 51 patients were invited to participate in the cohort study; 41 patients provided informed consent, and 40 patients were enrolled (eFigure in the Supplement). One participant was unable to enroll because of technical difficulties associated with the operation of the robotic system. The mean (SD) age of participants was 45.8 (2.7) years; 29 participants (72.5%) were female, and 11 participants (27.5%) were male (Table 1). A total of 22 participants (55.0%) were White, and 25 participants (62.5%) had attended college, received a college degree, or attended graduate school. All enrolled participants completed the quantitative assessment to measure their satisfaction and attitudes regarding their encounter with the robotic system.

**Table 1.** Characteristics of Participants in the Cohort Study

Characteristic	Participants, No. (%)
Total participants, No.	40
Age, mean (SD), y	45.8 (2.7)
Sex	
Male	11 (27.5)
Female	29 (72.5)
Race/ethnicity^a^	
White	22 (55.0)
Black or African American	7 (17.5)
Latino or Hispanic	9 (22.5)
Asian	2 (5.0)
Other^b^	1 (2.5)
Educational level	
<High school	5 (12.5)
High school graduate	5 (12.5)
Some college	12 (30.0)
College degree	11 (27.5)
Some graduate school	2 (5.0)
Trade school	2 (5.0)
Graduate degree	3 (7.5)

^a^ Participants could select more than 1 race/ethnicity.

^b^ Specific races and ethnicities included in this category were not specified.

In total, 37 participants (92.5%) reported being satisfied with the robotic system, and 34 participants (85.0%) were also satisfied with their interaction with the clinician who used the robotic system to facilitate the interview (Table 2). A total of 38 participants (95.0%) were satisfied with the video quality of the robotic system. Despite experiencing an ED environment that can be loud and chaotic, 35 participants (87.5%) reported that the on-board audio quality was satisfactory for understanding questions and interacting with the clinician.

**Table 2.** Satisfaction With the Mobile Robotic System Among Participants in the Cohort Study

Variable	Participants, No. (%)
Total participants, No.	40
Overall satisfaction with robotic system	
Dissatisfied	0
Neutral	3 (7.5)
Satisfied	37 (92.5)
Interaction with clinician using robotic system	
Dissatisfied	0
Neutral	6 (15.0)
Satisfied	34 (85.0)
Video quality of robotic system	
Dissatisfied	0
Neutral	2 (5.0)
Satisfied	38 (95.0)
Audio quality of robotic system	
Dissatisfied	2 (5.0)
Neutral	3 (7.5)
Satisfied	35 (87.5)
Interaction as satisfactory as in-person encounter	
Disagree	5 (12.5)
Neutral	2 (5.0)
Agree	33 (82.5)
Information provided by clinician using robotic system	
Dissatisfied	0
Neutral	3 (7.5)
Satisfied	37 (92.5)
Comfort interacting with clinician using a robotic system	
Uncomfortable	0
Neutral	5 (12.5)
Comfortable	35 (87.5)
Robotic system is acceptable to receive care	
Disagree	1 (2.5)
Neutral	5 (12.5)
Agree	34 (85.0)
Willing to interact with robotic system again	
Disagree	1 (2.5)
Neutral	2 (5.0)
Agree	37 (92.5)

Notably, 33 participants (82.5%) considered their robot-facilitated interaction with the clinician to be as satisfactory as a traditional in-person encounter, with 35 participants (87.5%) reporting that their clinician was able to provide adequate information that was understandable, despite the clinician not being physically present in the triage space. When asked about future health care–associated visits, 34 participants (85.0%) considered virtual care facilitated by a robotic system to be acceptable, and 37 participants (92.5%) reported that they would be willing to interact with a robotic system in the future.

## Discussion

The risk of SARS-CoV-2 infection and increased social distancing measures have changed the way in which in-person health care visits are conducted during the COVID-19 pandemic. The results of this study indicate that there is interest among the general public regarding acceptance of the use of robotic systems for patient interactions in the hospital, and this interest was reflected within our real-world pilot study of the use of a mobile robotic system to facilitate teletriage and patient interviews in the ED during the COVID-19 pandemic. These findings suggest that using a robotic system to facilitate contactless teletriage in the ED is feasible and acceptable, with implications for public health during the COVID-19 pandemic.

Our national survey results indicate that most individuals believe that robotic systems can be useful for in-hospital patient interactions, including performance of initial ED-based interviews, acquisition of contactless vital signs, basic testing for SARS-CoV-2 via nasal and oral swabs, resuscitation through placement of intravenous catheters, performance of phlebotomy, and potential assistance with tasks such as proning among patients who are critically ill. We expect that robotic systems can be developed to assist with these tasks, especially during periods in which more patients with potential COVID-19 present to the hospital.

Although robotic systems have been implemented in hospitals to deliver and replenish supplies, their use in facilitating human interaction has been limited.^25,26^ Some pilot studies have reported that using a robotic system for telerounding in inpatient units is feasible.^27,28^ Despite the feasibility of robotic systems, substantial barriers to expanding access and implementation in the hospital setting have been identified; these barriers are associated with technical support and unclear acceptance of these systems for use in clinical care.^29,30,31^ In the present cohort study, we were able to train emergency medical professionals in the operation of a robotic system and integrate the system into our existing telehealth platform to facilitate contactless triage interviews in the ED. Unlike inpatient settings, the ED setting presents distinct challenges with regard to navigating robotic systems through chaotic environments and interacting with patients in various locations.^32^ Although we experienced challenges in radio communication between the controller and the robotic system, we were able to overcome this barrier by identifying the potential interference of these radio bands through the use of communications packages required to control the robotic system. This approach allowed us to select an optimal suite of communications channels to reliably operate the robot in a radio-cluttered environment. Despite these challenges, participants were able to successfully engage with our robotic teletriage system, and 82.5% of participants considered this interaction to be equivalent in quality to an in-person interview. By designing a robotic platform and triage system that is acceptable to patients, we expect that we can continue to engage patients in the ED during periods, such as pandemics, when in-person visits are less likely to occur.

Robotic systems that facilitate contactless triage could have the potential to further reduce in-hospital SARS-CoV-2 transmission and conserve personal protective equipment. Minimizing human contact with individuals who have COVID-19 but are otherwise healthy may reduce the risk of in-hospital disease transmission and enable health care professionals at high risk of infection to safely interact with patients through teletriage. Furthermore, by using contactless systems to perform triage among individuals with low acuity, clinicians in the ED may be able to conserve resources by eliminating physical contact with these patients. In the context of regional increases in COVID-19, these incremental evaluations, which can be safely completed without the need for personal protective equipment, may help to improve the inventory of important materials in times of shortage or supply chain disruption. In addition, a robotic triage system may allow ED personnel the flexibility of screening individuals with lower acuity in a contactless manner while fulfilling the requirements of the Emergency Medical Treatment and Labor Act.^33^ Future work may consider approaches to maximize acceptance of robotic systems among patients, especially those who declined to participate in the present study.

### Limitations

This study has several limitations. First, although we used a complex approach for the national survey that comprised sample matching and weight adjustment methods that were previously validated,^34^ internet-based nonprobabilistic opt-in panels can have substantial biases, including the need for internet access and opt-in panel membership. Second, the national survey was administered through a national sampling platform consisting of individuals living in the US. Depending on their personal experiences with the pandemic, respondents’ attitudes toward robotic systems may have varied. In addition, the individuals enrolled in the survey study were predominantly White, with high educational levels. Third, the cohort study was conducted in the ED of a single large urban academic hospital. The experiences of using a complex robotic system such as ours may vary in other medical centers. Fourth, we did not collect demographic data on individuals who were approached for the study but declined to participate. This lack of data may have introduced selection bias into the cohort study.

Fifth, we used a highly agile mobile robotic system to facilitate telehealth tasks. The user response to other robotic systems may vary. Sixth, we decontaminated the robotic system using ethanol wipes, which may be time- and resource-intensive for personnel at many medical centers. Future iterations of a cleaning system may include an on-board automated function that can be remotely activated after a patient encounter as well as an UV radiation enclosure to permit sterilization during storage.

## Conclusions

The study’s results indicate that interaction with robotic systems to facilitate traditional in-person interviews in the ED is feasible and acceptable to patients. Several issues regarding the operation of these systems in a hospital setting warrant consideration. For example, findings from the national survey suggest that individuals find robotic systems useful in facilitating important hospital tasks that have traditionally been performed in person. This finding may inform the development of additional robotic systems that can minimize the exposure of health care professionals to individuals with COVID-19. Future iterations of robotic telehealth systems may include additional remote operators, such as individuals who have a higher risk of experiencing complications associated with COVID-19 or individuals recovering from COVID-19. These additional operators may be instrumental in conducting assessments of individuals with lower risk, as the operators will be able to work remotely as they recover from or minimize their own exposure to SARS-CoV-2. Cost-effectiveness studies of different robotic systems for various hospital-based tasks are warranted to help define the role and value of robotic systems in the context of the COVID-19 pandemic.

## Notes

Supplement. **eMethods.** National Survey Code Book **eTable 1.** Participant Demographic Characteristics From National Survey **eTable 2.** Participant Response Regarding Usefulness of Robotic Systems for Tasks in the Hospital in the National US Survey **eFigure.** Emergency Department Cohort Study Enrollment Characteristics
