# Supplementary material for: Assessment of the Acceptability and Feasibility of Using Mobile Robotic Systems for Patient Evaluation
Source: JAMA Netw Open. 2021 Mar 4;4(3):e210667. doi: 10.1001/jamanetworkopen.2021.0667 (PMC8058534; doi:10.1001/jamanetworkopen.2021.0667)
Supplement: Supplement. — eMethods. National Survey Code Book eTable 1. Participant Demographic Characteristics From National Survey eTable 2. Participant Response Regarding Usefulness of Robotic Systems for Tasks in the Hospital in the National US Survey eFigure. Emergency Department Cohort Study Enrollment Characteristics [file jamanetwopen-e210667-s001.pdf]

Supplementary Online Content

Chai PR, Dadabhoy FZ, Huang HW, et al. Assessment of the acceptability and feasibility of using mobile robotic systems for patient evaluation. *JAMA Netw Open*. 2021;4(3):e210667. doi:10.1001/jamanetworkopen.2021.0667

eMethods. National Survey Code Book

eTable 1. Participant Demographic Characteristics From National Survey

eTable 2. Participant Response Regarding Usefulness of Robotic Systems for Tasks in the Hospital in the National US Survey

eFigure. Emergency Department Cohort Study Enrollment Characteristics

This supplementary material has been provided by the authors to give readers additional information about their work.

© 2021 Chai PR et al. *JAMA Network Open*.

eMethods. National Survey Code Book

==

Project Code: MITU0032
Project Name: Robotics in Healthcare
Prepared for: Giovanni Traverso, Assistant Professor, MIT
Interviews: 1000
Field Period: August 18, 2020 - August 26, 2020
Project Manager: Alexander Marsolais <alexander.marsolais@yougov.com>

==

YouGov interviewed 1119 respondents who were then matched down to a sample of 1000 to produce the final dataset. The respondents were matched to a sampling frame on gender, age, race, and education. The frame was constructed by stratified sampling from the full 2017 American Community Survey (ACS) 1-year sample with selection within strata by weighted sampling with replacements (using the person weights on the public use file).

The matched cases were weighted to the sampling frame using propensity scores. The matched cases and the frame were combined and a logistic regression was estimated for inclusion in the frame. The propensity score function included age, gender, race/ethnicity, years of education, and region. The propensity scores were grouped into deciles of the estimated propensity score in the frame and post-stratified according to these deciles.

The weights were then post-stratified on 2016 Presidential vote choice, and a four-way stratification of gender, age (4-categories), race (4-categories), and education (4-categories), to produce the final weight.

==

Variable List

==

caseid Case ID
weight Gen Pop Weight
consent consent

1. Q1_1 Q1--I would feel uneasy if robots really had

emotions.

2. Q1_2 Q1 -- Something bad might happen if robots
3. developed into living beings.
4.
5. Q1_3 Q1 -- I would feel relaxed talking with robots.
6.
7. Q1_4 Q1 -- I would feel uneasy if I was given a job
8. where I had to use robots.
9.
10. Q1_5 Q1 -- If robots had emotions, I would be able to
11. make friends with them.
12.
13. Q1_6 Q1 -- I feel comforted being with robots that
14. have emotions.
15.
16. Q1_7 Q1 -- The word “robot” means nothing to me.
17.
18. Q1_8 Q1 -- I would feel nervous operating a robot in
19. front of other people.

© 2021 Chai PR et al. *JAMA Network Open*.

20.
21. Q1_9 Q1 -- I would hate the idea that robots or
22. artificial intelligences were making judgments
23. about things.
24.
25. Q1_10 Q1 -- I would feel very nervous just standing in
26. front of a robot.
27.
28. Q1_11 Q1 -- I feel that if I depend on robots too much,
29. something bad might happen.
30.
31. Q1_12 Q1 -- I would feel paranoid talking with a robot.
32.
33. Q1_13 Q1 -- I am concerned that robots would be a bad
34. influence on children.
35.
36. Q1_14 Q1 -- I feel that in the future society will be
37. dominated by robots.
38.
39. Q1_15 Q1 -- It is acceptable to receive care through
40. robots for my own safety.
41.
42. Q1_16 Q1 -- It is acceptable to receive care through
43. robots for the safety of healthcare workers.
44.

1. Q2_1 Q2 -- Helping a patient get out of a chair
2.
3. Q2_2 Q2 -- Detecting falls and calling for help
4.
5. Q2_3 Q2 -- Vital signs assessment e.g. taking a
6. patient’s blood pressure, temperature, breathing
7. rate, oxygen level or heart rate
8.

Q2_4

Q2_5
Q2_6
Q2_7
Q2_8
Q2_9

Q2_10

Q2_11
Q2_12
Q2_13
Q2_14
Q2_15
Q3_1
Q3_2
Q3_3

Q3_4

[illegible]

Q3_5
Q3_6
Q3_7
Q3_8
Q3_9

Q3_10

Q3_11
Q3_12
Q3_13
Q3_14
Q3_15
Q4
birthyr
gender
race
educ
marstat
child18
employ
faminc_new
pid3
pid7
presvote16post
inputstate
region
votereg
ideo5
newsint
religpew
religpew_protestant
pew_churatd
pew_bornagain
pew_religimp
pew_prayer
genhealth
insurtype_1
insurtype_2
insurtype_3
Q2 -- Helping make phone or video calls to a patient's family member
Q2 -- Dementia care e.g. telling the day/time
Q2 -- Walking assistance
Q2 -- Responding to call for assistance
Q2 -- Helping move or turn a patient in their bed
Q2 -- Helping a patient with their personal care (e.g., showering)
Q2 -- Facilitating a telemedicine interview with a patient's doctor or nurse
Q2 -- Performing nasal or oral swabs on a patient
Q2 -- Drawing blood from a patient for tests
Q2 -- Starting an intravenous (IV) line in a patient
Q2 -- Administer oral medications for a patient
Q2 -- Administer intravenous (IV) medications
Q3 -- Helping a patient get out of a chair
Q3 -- Detecting falls and calling for help
Q3 -- Vital signs assessment e.g. taking a

© 2021 Chai PR et al. *JAMA Network Open*.

patient’s blood pressure, temperature, breathing
rate, oxygen level or heart rate
Q3 -- Helping make phone or video calls to a
patient’s family member
Q3 -- Dementia care e.g. telling the day/time
Q3 -- Walking assistance
Q3 -- Responding to call for assistance
Q3 -- Helping move or turn a patient in their bed
Q3 -- Helping a patient with their personal care
(e.g., showering)
Q3 -- Facilitating a telemedicine interview with
a patient’s doctor or nurse
Q3 -- Performing nasal or oral swabs on a patient
Q3 -- Drawing blood from a patient for tests
Q3 -- Starting an intravenous (IV) line in a
patient
Q3 -- Administer oral medications for a patient
Q3 -- Administer intravenous (IV) medications
Q4
Birth Year
Gender
Race
Education
Marital Status
Children under age 18 in household
Employment Status
Family income
3 point party ID
7 point Party ID
2016 President Vote Post Election
State of Residence
Region
Voter Registration Status
Ideology
Political Interest
Religion
Protestant Church
Church attendance (Pew version)
Born Again (Pew version)
Importance of religion (Pew version)
Frequency of Prayer (Pew version)
General health condition
I have health insurance that is provided through
my or a family member's employer, union, school,
trade group or professional association
I have health insurance provided through Medicare
I have health insurance that I or a family member
purchased directly from the heath insurance
company or HMO, not through an employer, union,
insurtype_4
insurtype_5
insurtype_99
pp_smoke100
starttime
endtime
school, trade group or professional association
I have health insurance through the military or
through VA Benefits
I have health insurance that is provided through
a government heath program, such as Medicaid

© 2021 Chai PR et al. *JAMA Network Open*.

Not applicable - I do not have health insurance
Smoking history
Questionnaire Start Time
Questionnaire End Time

Verbatims

==

employ_t
pid3_t
inputzip
Employment Status - open
3 point party ID - other
Zip Input

Variable map and codebook

==

Name: caseid
Description: Case ID
Numeric Variable - no categories
answered : 1000

==

Name: weight
Description: Gen Pop Weight
Numeric Variable - no categories
answered : 1000

==

Name: consent
Description: consent

Count	Code	Label
1000	1	Yes
0	2	No, please take me to another survey

==

Name: Q1_1
Description: Q1 -- I would feel uneasy if robots really had emotions.

Count	Code	Label
54	1	Strongly disagree
120	2	Disagree
230	3	Neither agree nor disagree
367	4	Agree
229	5	Strongly agree

==

Name: Q1_2
Description: Q1 -- Something bad might happen if robots developed into living

beings.

Count	Code	Label
35	1	Strongly disagree
83	2	Disagree
204	3	Neither agree nor disagree
383	4	Agree
295	5	Strongly agree

==

Name: Q1_3
Description: Q1 -- I would feel relaxed talking with robots.

Count	Code	Label
107	1	Strongly disagree

© 2021 Chai PR et al. *JAMA Network Open*.

211 2 Disagree
307 3 Neither agree nor disagree
301 4 Agree
74 5 Strongly agree

==

Name: Q1_4
Description: Q1 -- I would feel uneasy if I was given a job where I had to

use robots.

Count	Code	Label
152	1	Strongly disagree
386	2	Disagree
260	3	Neither agree nor disagree
128	4	Agree
74	5	Strongly agree

==

Name: Q1_5
Description: Q1 -- If robots had emotions, I would be able to make friends

with them.

Count	Code	Label
135	1	Strongly disagree
211	2	Disagree
363	3	Neither agree nor disagree
233	4	Agree
58	5	Strongly agree

==

Name: Q1_6
Description: Q1 -- I feel comforted being with robots that have emotions.

Count	Code	Label
196	1	Strongly disagree
295	2	Disagree
322	3	Neither agree nor disagree
140	4	Agree
47	5	Strongly agree

==

Name: Q1_7
Description: Q1 -- The word “robot” means nothing to me.

Count	Code	Label
135	1	Strongly disagree
406	2	Disagree
304	3	Neither agree nor disagree
123	4	Agree
32	5	Strongly agree

==

Name: Q1_8
Description: Q1 -- I would feel nervous operating a robot in front of other

people.

Count Code Label
----- ---- -----

© 2021 Chai PR et al. *JAMA Network Open*.

145 1 Strongly disagree
392 2 Disagree
249 3 Neither agree nor disagree
158 4 Agree
56 5 Strongly agree
==
Name: Q1_9
Description: Q1 -- I would hate the idea that robots or artificial intelligences were making judgments about things.

Count	Code	Label
-----	----	-----
49	1	Strongly disagree
190	2	Disagree
257	3	Neither agree nor disagree
309	4	Agree
195	5	Strongly agree

==
Name: Q1_10
Description: Q1 -- I would feel very nervous just standing in front of a

robot.

Count	Code	Label
-----	----	-----
176	1	Strongly disagree
403	2	Disagree
223	3	Neither agree nor disagree
136	4	Agree
62	5	Strongly agree

==
Name: Q1_11
Description: Q1 -- I feel that if I depend on robots too much, something bad might happen.

Count	Code	Label
-----	----	-----
53	1	Strongly disagree
217	2	Disagree
252	3	Neither agree nor disagree
340	4	Agree
137	5	Strongly agree

1 8 skipped

==
Name: Q1_12
Description: Q1 -- I would feel paranoid talking with a robot.

Count	Code	Label
-----	----	-----
104	1	Strongly disagree
383	2	Disagree
264	3	Neither agree nor disagree
174	4	Agree
75	5	Strongly agree

==
Name: Q1_13
Description: Q1 -- I am concerned that robots would be a bad influence on

children.

© 2021 Chai PR et al. *JAMA Network Open*.

Count	Code	Label
-----	----	-----
103	1	Strongly disagree
302	2	Disagree
319	3	Neither agree nor disagree
184	4	Agree
92	5	Strongly agree

==

Name: Q1_14

Description: Q1 -- I feel that in the future society will be dominated by

robots.

Count	Code	Label
-----	----	-----
72	1	Strongly disagree
243	2	Disagree
307	3	Neither agree nor disagree
293	4	Agree
85	5	Strongly agree

==

Name: Q1_15

Description: Q1 -- It is acceptable to receive care through robots for my own

safety.

Count	Code	Label
-----	----	-----
72	1	Strongly disagree
128	2	Disagree
315	3	Neither agree nor disagree
401	4	Agree
84	5	Strongly agree

==

Name: Q1_16

Description: Q1 -- It is acceptable to receive care through robots for the safety of healthcare workers.

Count	Code	Label
-----	----	-----
69	1	Strongly disagree
103	2	Disagree
242	3	Neither agree nor disagree
465	4	Agree
121	5	Strongly agree

==

Name: Q2_1

Description: Q2 -- Helping a patient get out of a chair

Count Code Label

----- ---- -----

47 1

49 2

106 3

423 4

375 5

Extremely useless

Somewhat useless

© 2021 Chai PR et al. *JAMA Network Open*.

Neither useful nor useless
Somewhat useful
Extremely useful
==
Name: Q2_2
Description: Q2 -- Detecting falls and calling for help
Count Code Label
----- ---- -----

24 1 29 2 79 3

312 4 556 5

Extremely useless
Somewhat useless
Neither useful nor useless
Somewhat useful
Extremely useful
==
Name: Q2_3
Description: Q2 -- Vital signs assessment e.g. taking a patient's blood
pressure, temperature, breathing rate, oxygen level or heart

rate

Count Code Label
----- ---- -----

46 1

62 2
129 3
350 4
413 5
Extremely useless
Somewhat useless
Neither useful nor useless
Somewhat useful
Extremely useful
==
Name: Q2_4
Description: Q2 -- Helping make phone or video calls to a patient's family

member

Count Code Label
----- ---- -----

57 1

90 2
131 3
368 4
354 5
Extremely useless
Somewhat useless

© 2021 Chai PR et al. *JAMA Network Open*.

Neither useful nor useless
Somewhat useful
Extremely useful
==
Name: Q2_5
Description: Q2 -- Dementia care e.g. telling the day/time
Count Code Label
----- ---- -----

50. 50 1
51. 51 2

147 3
386 4
366 5
Extremely useless
Somewhat useless
Neither useful nor useless
Somewhat useful
Extremely useful
==
Name: Q2_6
Description: Q2 -- Walking assistance
Count Code Label
----- ---- -----

39 1 49 2 98 3

403 4 411 5

Extremely useless
Somewhat useless
Neither useful nor useless
Somewhat useful
Extremely useful
==
Name: Q2_7
Description: Q2 -- Responding to call for assistance
Count Code Label
----- ---- -----

45 1

79 2
131 3
360 4
385 5
Extremely useless
Somewhat useless
Neither useful nor useless
Somewhat useful
Extremely useful
==
Name: Q2_8
Description: Q2 -- Helping move or turn a patient in their bed
Count Code Label
----- ---- -----

© 2021 Chai PR et al. *JAMA Network Open*.

59 1

63 2 136 3

Extremely useless
Somewhat useless
Neither useful nor useless
371 4 Somewhat useful
371 5 Extremely useful
==
Name: Q2_9
Description: Q2 -- Helping a patient with their personal care (e.g.,

showering)

Count Code Label
----- ---- -----
88 1
133 2
182 3
327 4
270 5
Extremely useless
Somewhat useless
Neither useful nor useless
Somewhat useful
Extremely useful
==
Name: Q2_10
Description: Q2 -- Facilitating a telemedicine interview with a patient's
doctor or nurse
Count Code Label
----- ---- -----

68 1

94 2
178 3
373 4
287 5
Extremely useless
Somewhat useless
Neither useful nor useless
Somewhat useful
Extremely useful
==
Name: Q2_11
Description: Q2 -- Performing nasal or oral swabs on a patient
Count Code Label
----- ---- -----
120 1
168 2
213 3
307 4
192 5
Extremely useless
Somewhat useless
Neither useful nor useless

© 2021 Chai PR et al. *JAMA Network Open*.

Somewhat useful
Extremely useful
==
Name: Q2_12
Description: Q2 -- Drawing blood from a patient for tests
Count Code Label
----- ---- -----
170 1
197 2
217 3
249 4
167 5
Extremely useless
Somewhat useless
Neither useful nor useless
Somewhat useful
Extremely useful
==
Name: Q2_13
Description: Q2 -- Starting an intravenous (IV) line in a patient
Count Code Label
----- ---- -----
190 1
210 2
213 3
228 4
159 5
Extremely useless
Somewhat useless
Neither useful nor useless
Somewhat useful
Extremely useful
==
Name: Q2_14
Description: Q2 -- Administer oral medications for a patient
Count Code Label
----- ---- -----

115. 115 1
116. 116 2

197 3
358 4
214 5
Extremely useless
Somewhat useless
Neither useful nor useless
Somewhat useful
Extremely useful
==
Name: Q2_15
Description: Q2 -- Administer intravenous (IV) medications
Count Code Label
----- ---- -----
168 1
187 2
216 3
250 4
179 5
© 2021 Chai PR et al. *JAMA Network Open*.

Extremely useless
Somewhat useless
Neither useful nor useless
Somewhat useful
Extremely useful
==
Name: Q3_1
Description: Q3 -- Helping a patient get out of a chair
Count Code Label
----- ---- -----

49 1

57 2
113 3
358 4
423 5
Extremely useless
Somewhat useless
Neither useful nor useless
Somewhat useful
Extremely useful
==
Name: Q3_2
Description: Q3 -- Detecting falls and calling for help
Count Code Label
----- ---- -----

37 1 39 2 89 3

347 4 488 5

Extremely useless
Somewhat useless
Neither useful nor useless
Somewhat useful
Extremely useful
==
Name: Q3_3
Description: Q3 -- Vital signs assessment e.g. taking a patient's blood pressure, temperature, breathing rate, oxygen level or heart rate
Count Code Label
----- ---- -----

76 1

62 2
123 3
336 4
403 5
Extremely useless
Somewhat useless
Neither useful nor useless
Somewhat useful
Extremely useful
==
Name: Q3_4

© 2021 Chai PR et al. *JAMA Network Open*.

Description: Q3 -- Helping make phone or video calls to a patient's family

member

Count Code Label
----- ---- -----

571 Extremely useless 832 Somewhat useless

136 3
353 4
370 5
Neither useful nor useless
Somewhat useful
Extremely useful

1 8 skipped

==
Name: Q3_5
Description: Q3 -- Dementia care e.g. telling the day/time
Count Code Label
----- ---- -----

47 1

68 2
149 3
357 4
379 5
Extremely useless
Somewhat useless
Neither useful nor useless
Somewhat useful
Extremely useful
==
Name: Q3_6
Description: Q3 -- Walking assistance
Count Code Label
----- ---- -----

53 1

51 2
109 3
373 4
414 5
Extremely useless
Somewhat useless
Neither useful nor useless
Somewhat useful
Extremely useful
==
Name: Q3_7
Description: Q3 -- Responding to call for assistance
Count Code Label
----- ---- -----

© 2021 Chai PR et al. *JAMA Network Open*.

50 1

69 2
120 3
364 4
397 5
Extremely useless
Somewhat useless
Neither useful nor useless
Somewhat useful
Extremely useful
==
Name: Q3_8
Description: Q3 -- Helping move or turn a patient in their bed
Count Code Label
----- ---- -----

64 1

91 2
136 3
331 4
378 5
Extremely useless
Somewhat useless
Neither useful nor useless
Somewhat useful
Extremely useful
==
Name: Q3_9
Description: Q3 -- Helping a patient with their personal care (e.g.,

showering)

Count Code Label
----- ---- -----
102 1
146 2
158 3
311 4
283 5
Extremely useless
Somewhat useless
Neither useful nor useless
Somewhat useful
Extremely useful
==
Name: Q3_10
Description: Q3 -- Facilitating a telemedicine interview with a patient's
doctor or nurse
Count Code Label
----- ---- -----

77 1

90 2
168 3
351 4

© 2021 Chai PR et al. *JAMA Network Open*.

314 5
Extremely useless
Somewhat useless
Neither useful nor useless
Somewhat useful
Extremely useful
==
Name: Q3_11
Description: Q3 -- Performing nasal or oral swabs on a patient
Count Code Label
----- ---- -----
116 1
163 2
195 3
287 4
239 5
Extremely useless
Somewhat useless
Neither useful nor useless
Somewhat useful
Extremely useful
==
Name: Q3_12
Description: Q3 -- Drawing blood from a patient for tests
Count Code Label
----- ---- -----
152 1
194 2
203 3
236 4
215 5
Extremely useless
Somewhat useless
Neither useful nor useless
Somewhat useful
Extremely useful
==
Name: Q3_13
Description: Q3 -- Starting an intravenous (IV) line in a patient
Count Code Label
----- ---- -----

167 1

198. 198 2
199. 199 3

228 4 208 5

Extremely useless
Somewhat useless
Neither useful nor useless
Somewhat useful
Extremely useful
==
Name: Q3_14
Description: Q3 -- Administer oral medications for a patient
Count Code Label

© 2021 Chai PR et al. *JAMA Network Open*.

----- ---- -----
108 1
106 2
182 3
331 4
273 5
Extremely useless
Somewhat useless
Neither useful nor useless
Somewhat useful
Extremely useful
==
Name: Q3_15
Description: Q3 -- Administer intravenous (IV) medications
Count Code Label
----- ---- -----
155 1
183 2
194 3
Extremely useless
Somewhat useless
Neither useful nor useless
246 4 Somewhat useful
222 5 Extremely useful
==
Name: Q4
Description: Q4
Count Code Label
----- ---- -----
99 1 Totally unacceptable
100 2 Unacceptable
213 3 Neither unacceptable nor acceptable
396 4 Acceptable
192 5 Totally acceptable
==
Name: birthyr
Description: Birth Year
Numeric Variable - no categories
answered : 1000
==
Name: gender
Description: Gender
Count Code Label
----- ---- -----
465 1 Male
535 2 Female
==
Name: race
Description: Race
Count Code Label
----- ---- -----
719 1 White
86 2 Black
103 3 Hispanic
40 4 Asian
9 5 Native American
17 6 Mixed
25 7 Other
1 8 Middle Eastern
==
© 2021 Chai PR et al. *JAMA Network Open*.

Name: educ
Description: Education

Count Code Label ----- ---- ----- 36 1NoHS

Count	Code	Label
287	2	High school graduate
235	3	Some college
106	4	2-year
221	5	4-year
115	6	Post-grad

==
Name: marstat
Description: Marital Status

Count	Code	Label
-----	----	-----
465	1	Married
21	2	Separated
112	3	Divorced
66	4	Widowed
292	5	Never married

446 Domestic / civil partnership

==
Name: child18
Description: Children under age 18 in household

Count	Code	Label
-----	----	-----
237	1	Yes

763 2No

==
Name: employ
Description: Employment Status

Count	Code	Label
-----	----	-----
336	1	Full-time
104	2	Part-time
39	3	Temporarily laid off
82	4	Unemployed
205	5	Retired
92	6	Permanently disabled
72	7	Homemaker
50	8	Student
20	9	Other

==
Name: faminc_new
Description: Family income

Count	Code	Label
-----	----	-----

73 1

78	2
104	3
94	4

© 2021 Chai PR et al. *JAMA Network Open*.

62 5
79 6
73 7

66. 66 8
67. 67 9

55 10
54 11
31 12
13 13
12 14
4 15
6 16
129 97
Less than $10,000
$10,000 - $19,999
$20,000 - $29,999
$30,000 - $39,999
$40,000 - $49,999
$50,000 - $59,999
$60,000 - $69,999
$70,000 - $79,999
$80,000 - $99,999
$100,000 - $119,999
$120,000 - $149,999
$150,000 - $199,999
$200,000 - $249,999
$250,000 - $349,999
$350,000 - $499,999
$500,000 or more
Prefer not to say
==
Name: pid3
Description: 3 point party ID

Count	Code	Label
362	1	Democrat
229	2	Republican
301	3	Independent

58 4 Other
50 5 Notsure

==
Name: pid7
Description: 7 point Party ID

Count	Code	Label
253	1	
109	2	
106	3	
160	4	Independent

96 5

64 6

© 2021 Chai PR et al. *JAMA Network Open*.

165 7
47 8
0 9

Lean Republican
Not very strong Republican Strong Republican
Notsure
Don'tknow

Strong Democrat
Not very strong Democrat
Lean Democrat
==
Name: presvote16post
Description: 2016 President Vote Post Election
Count Code Label
----- ---- -----
327 1
268 2
31 3
24 4
5 5
Hillary Clinton
Donald Trump
Gary Johnson
Jill Stein
Evan McMullin
18 6 Other
327 7 Did not vote for President
==
Name: inputstate
Description: State of Residence

Count	Code	Label
-----	----	-----
19	1	Alabama
1	2	Alaska
27	4	Arizona
16	5	Arkansas
92	6	California
16	8	Colorado
4	9	Connecticut
1	10	Delaware
4	11	District of Columbia
74	12	Florida
20	13	Georgia
3	15	Hawaii
10	16	Idaho
38	17	Illinois
24	18	Indiana

9 19 Iowa

8	20	Kansas
11	21	Kentucky
14	22	Louisiana
3	23	Maine
13	24	Maryland
17	25	Massachusetts
25	26	Michigan

© 2021 Chai PR et al. *JAMA Network Open*.

16 27 Minnesota
3 28 Mississippi
18 29 Missouri

6 30 Montana

7 31 Nebraska
19 32 Nevada
2 33
41 34
7 35
61 36
32 37
4 38

New Hampshire New Jersey
New Mexico NewYork
North Carolina North Dakota

31 39 Ohio
7 40 Oklahoma
18 41 Oregon
52 42 Pennsylvania

0 44 18 45 5 46

Rhode Island
South Carolina
South Dakota
30 47 Tennessee
75 48 Texas

8 49 Utah

2 50 Vermont
28 51 Virginia
30 53 Washington
7 54 West Virginia
20 55 Wisconsin
4 56 Wyoming
0 60 American Samoa
0 64 Federated States of Micronesia
0 66 Guam
0 68 Marshall Islands
0 69 Northern Mariana Islands
0 70 Palau
0 72
0 74
0 78
0 81 Alberta
0 82 British Columbia
0 83 Manitoba
Puerto Rico
U.S. Minor Outlying Islands
Virgin Islands
0 84 New Brunswick
0 85 Newfoundland

© 2021 Chai PR et al. *JAMA Network Open*.

0	86	Northwest Territories
0	87	Nova Scotia
0	88	Nunavut
0	89	Ontario
0	90	Prince Edward Island
0	91	Quebec
0	92	Saskatchewan
0	93	Yukon Territory
0	99	Not in the U.S. or Canada

==

Name: region

Description: Region

Count	Code	Label
-----	----	-----
182	1	Northeast
205	2	Midwest
372	3	South
241	4	West

==

Name: votereg

Description: Voter Registration Status

Count	Code	Label
-----	----	-----
848	1	Yes

130 2No

22 3 Don't know

==

Name: ideo5

Description: Ideology

Count	Code	Label
-----	----	-----
118	1	Very liberal
166	2	Liberal
318	3	Moderate
189	4	Conservative
119	5	Very conservative

90 6 Notsure

==

Name: newsint

Description: Political Interest

Count Code Label

----- ---- -----

513 1

272 2

106 3

67 4 42 7

Most of the time

Some of the time

Only now and then

Hardly at all

Don't know

==

© 2021 Chai PR et al. *JAMA Network Open*.

Name: religpew
Description: Religion

Count	Code	Label
313	1	Protestant
180	2	Roman Catholic
12	3	Mormon
7	4	Eastern or Greek Orthodox
30	5	Jewish
18	6	Muslim
13	7	Buddhist
1	8	Hindu
62	9	Atheist
65	10	Agnostic
220	11	Nothing in particular
79	12	Something else

==

Name: religpew_protestant
Description: Protestant Church

Count	Code	Label
114	1	Baptist
41	2	Methodist
74	3	Nondenominational or Independent Church
39	4	Lutheran
22	5	Presbyterian
24	6	Pentecostal
13	7	Episcopalian
16	8	Church of Christ or Disciples of Christ
8	9	Congregational or United Church of Christ
3	10	Holiness
4	11	Reformed
5	12	Adventist
9	13	Jehovah's Witness
19	90	Something else
609	98	skipped

==

Name: pew_churatd
Description: Church attendance (Pew version)

Count Code Label
----- ---- -----

72 1 174 2 66 3

More than once a week
Once a week
Once or twice a month

Count	Code	Label
131	4	A few times a year
195	5	Seldom
341	6	Never

217 Don't know

==

Name: pew_bornagain
Description: Born Again (Pew version)

Count	Code	Label
300	1	Yes

© 2021 Chai PR et al. *JAMA Network Open*.

700 2No

==
Name: pew_religimp
Description: Importance of religion (Pew version)
Count Code Label
----- ---- -----
393 1
227 2
144 3
236 4
Very important
Somewhat important
Not too important
Not at all important
==
Name: pew_prayer
Description: Frequency of Prayer (Pew version)
Count Code Label
----- ---- -----
290 1
124 2
121 3

25 4

Several times a day
Once a day
A few times a week
Once a week
57 5
134 6 Seldom
209 7 Never
A few times a month
40 8 Don't know
==
Name: genhealth
Description: General health condition
Count Code Label
----- ---- -----
137 1 Excellent
343 2 Very good
313 3 Good
167 4 Fair

40 5 Poor

==
Name: insurtype_1
Description: I have health insurance that is provided through my or a family member's employer, union, school, trade group or professional association
Count Code Label
----- ---- -----
418 1 selected
582 2 not selected
==
Name: insurtype_2

© 2021 Chai PR et al. *JAMA Network Open*.

Description: I have health insurance provided through Medicare
Count Code Label
----- ---- -----
248 1 selected
752 2 not selected
==
Name: insurtype_3
Description: I have health insurance that I or a family member purchased
directly from the heath insurance company or HMO, not through an
employer, union, school, trade group or professional association
Count Code Label
----- ---- -----
104 1 selected
896 2 not selected
==
Name: insurtype_4
Description: I have health insurance through the military or through VA

Benefits

Count Code Label
----- ---- -----
47 1 selected
953 2 not selected
==
Name: insurtype_5
Description: I have health insurance that is provided through a government
heath program, such as Medicaid
Count Code Label
----- ---- -----
152 1 selected
848 2 not selected
==
Name: insurtype_99
Description: Not applicable - I do not have health insurance
Count Code Label
----- ---- -----
119 1 selected
881 2 not selected
==
Name: pp_smoke100
Description: Smoking history
Count Code Label
----- ---- -----
433 1 Yes

567 2No

Date format variables
==
Name: starttime
Description: Questionnaire Start Time
DateTime variable - no categories
==
Name: endtime
Description: Questionnaire End Time
DateTime variable - no categories

© 2021 Chai PR et al. *JAMA Network Open*.

e**Table 1**. Participant Demographic Characteristics From National Survey

Demographic	N= 1000
Age (years)	48.7 +/- 17.0
Sex	
Male	465 (46.5%)
Female	535 (53.4%)
Geography (Regions by Census)	
Northeast	182 (18.2%)
Midwest	205 (20.5%)
South	372 (37.2%)
West	241 (24.1%)
Ethnicity	
White or Caucasian	719 (71.9%)
Black or African American	86 (8.6%)
Latino or Hispanic	103 (10.3%)
Asian	40 (4.0%)
Native American	9 (0.90%)
Middle Eastern	1 (0.10%)
Mixed	17 (1.7%)
Other	25 (2.5%)
Education	
No high school	36 (3.6%)
High school graduate	287 (28.7%)
Some college	235 (23.5%)
2-year college degree	106 (10.6%)
4-year college degree	221 (22.1%)
Post-graduate	115 (11.5%)

© 2021 Chai PR et al. *JAMA Network Open*.

eTable 2. Participant Response Regarding Usefulness of Robotic Systems for Tasks in the Hospital in the National US Survey

	Usefulness of Robotic Systems for hospital tasks	Usefulness of Robotic Systems for hospital tasks in context of COVID-19	p-value
Facilitating telemedicine interview with physician or nurse			0.16
1-Extremely Useless	68 (6.8%)	77 (7.7%)	
2-Somewhat Useless	94 (9.4%)	90 (9.0%)	
3-Neither Useful nor Useless	178 (17.8%)	168 (16.8%),	
4- Somewhat Useful	373 (37.3%)	351 (35.1%)	
5-Extremely Useful	287 (28.7%)	314 (31.4%)	
Median Score	4	4	
IQR (p75-p25)	(5-3)=2	(5-3)=2	
Acquisition of contactless vital signs			0.0003
1-Extremely Useless	46 (4.6%)	76 (7.6%)	
2-Somewhat Useless	62 (6.2%)	62 (6.2%)	
3- Neither Useful nor Useless	129 (12.9%)	123 (12.3%)	
4- Somewhat Useful	350 (35.0%)	336 (33.6%)	
5-Extremely Useful	413 (41.3%)	403 (40.3%)	
Median Score	4	4	
IQR (p75-p25)	(5-4) =1	(5-3) =2	
Obtaining nasal or oral swabs			0.002
1-Extremely Useless	120 (12.0%)	116 (11.6%)	
2-Somewhat Useless	168 (16.8%)	163 (16.3%)	
3- Neither Useful nor Useless	213 (21.3%)	195 (19.5%)	
4- Somewhat Useful	307 (30.7%)	287 (28.7%)	
5-Extremely Useful	192 (19.2%)	239 (23.9%)	
Median Score	3	4	
IQR (p75-p25)	(4-2) =2	(4-2) =2	
Placing an intravenous catheter			<0.001
1-Extremely Useless	190 (19.0%)	167 (16.7%)	
2-Somewhat Useless	210 (21.0%)	198 (19.8%)	
3- Neither Useful nor Useless	213 (21.3%)	199 (19.9%)	
4- Somewhat Useful	228 (22.8%)	228 (22.8%)	
5-Extremely Useful	159 (15.9%)	208 (20.8%)	

© 2021 Chai PR et al. *JAMA Network Open*.

Median Score<br>*IQR (p75-p25)*	3<br>(4-2) =2	3<br>(4-2) =2	
Performing phlebotomy			<0.001
1-Extremely Useless	170 (17.0%)	152 (15.2%)	
2-Somewhat Useless	197 (19.7%)	194 (19.4%)	
3- Neither Useful nor Useless	217 (21.7%)	203 (20.3%)	
4- Somewhat Useful	249 (24.9%)	236 (23.6%)	
5-Extremely Useful	167 (16.7%)	215 (21.5%)	
Median Score<br>*IQR (p75-p25)*	3<br>(4-2) =2	3<br>(4-2) =2	
Helping position or turn a patient in bed			0.04
1-Extremely Useless	59 (5.9%)	64 (6.4%)	
2-Somewhat Useless	63 (6.3%)	91 (9.1%)	
3- Neither Useful nor Useless	136 (13.6%)	136 (13.6%)	
4- Somewhat Useful	371 (37.1%)	331 (33.1%)	
5-Extremely Useful	371 (37.1%)	378 (37.8%)	
Median score<br>*IQR (p75-p25)*	4<br>(5-3) =2	4<br>(5-3) =2	
N=1000<br>**Survey scores using a 5-Point Likert Scale*			

© 2021 Chai PR et al. *JAMA Network Open*.

eFigure. Emergency Department Cohort Study Enrollment Characteristics

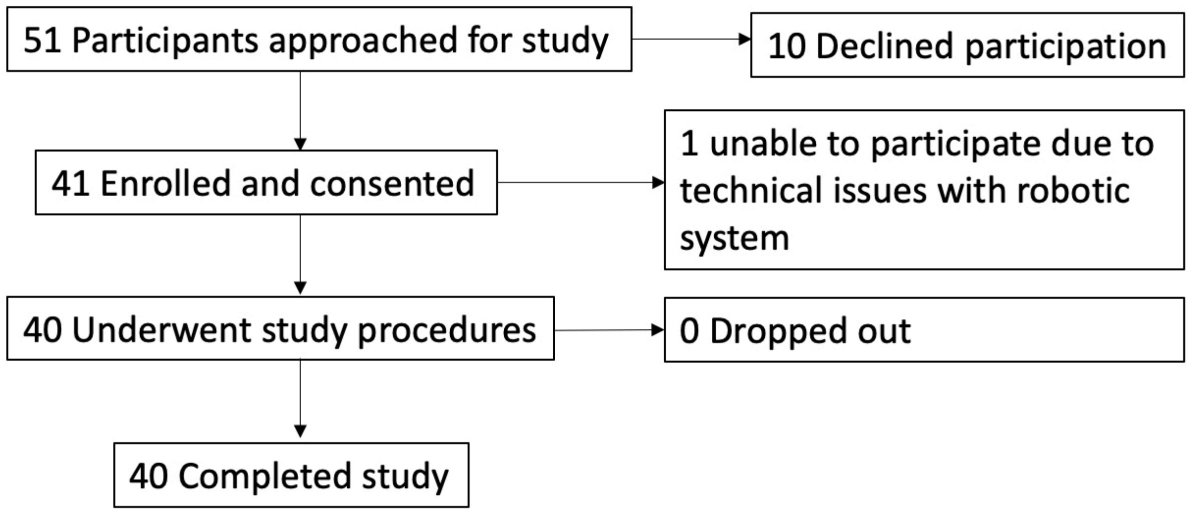

© 2021 Chai PR et al. *JAMA Network Open*.
